# 原发中枢神经系统大B细胞淋巴瘤临床病理特点及生存分析

**DOI:** 10.3760/cma.j.cn121090-20231126-00278

**Published:** 2024-05

**Authors:** 起帆 许, 容 沈, 一格 沈, 怡文 曹, 樱 钱, 彭鹏 许, 澍 程, 黎 王, 维莅 赵

**Affiliations:** 上海交通大学医学院附属瑞金医院血液科，医学基因组学国家重点实验室，上海血液学研究所，上海 200025 Department of Haematology, State Key Laboratory of Medical Genomics, Shanghai Institute of Haematology, Ruijin Hospital Affiliated to Shanghai Jiao Tong University School of Medicine, Shanghai 200025, China

**Keywords:** 淋巴瘤，大B细胞，弥漫性, 中枢神经系统, 布鲁顿酪氨酸激酶, 突变, 预后, Lymphoma, large B-cell, diffuse, Central nervous system, Bruton tyrosine kinase, Mutation, Prognosis

## Abstract

**目的:**

回顾性分析原发中枢神经系统大B细胞淋巴瘤（PCNSLBCL）患者的临床、病理特征、疗效、生存和预后情况。

**方法:**

回顾性分析上海交通大学医学院附属瑞金医院自2010年10月至2022年11月收治的70例PCNSLBCL患者的临床和病理资料，采用Kaplan-Meier法及Log-rank检验进行生存分析以及Cox比例风险模型进行预后分析。

**结果:**

70例PCNSLBCL患者接受一线诱导治疗后49例（70.0％）评价为完全缓解（CR），4例（5.7％）评价为部分缓解，客观缓解率为75.7％。2年无进展生存（PFS）率55.8％，中位PFS期为35.9个月；2年总生存（OS）率79.1％，中位OS期未达到。一线诱导CR后，接受auto-HSCT的患者累积复发率（CIR）低于未接受任何巩固治疗的患者（*P*＝0.032）；口服小分子药物维持的患者2年PFS率为84.4％，中位PFS期为79.5个月，无巩固治疗患者2年PFS率为54.4％，中位PFS期为35.9个月，差异有统计学意义（*P*＝0.038）。多因素分析显示纪念斯隆-凯特琳肿瘤中心（MSKCC）预后评分3类是影响PCNSLBCL患者OS的独立预后不良因素（*HR*＝3.127，95％ *CI* 1.057～9.253，*P*＝0.039）。

**结论:**

一线诱导治疗CR的PCNSLBCL患者接受auto-HSCT巩固治疗可降低CIR，口服小分子药物维持治疗可延长PFS期。MSKCC预后评分3类与PCNSLBCL患者较差的OS相关。

## Abstract (en)

**Objective:**

To retrospectively analyze the clinical and pathologic characteristics, response to treatment, survival, and prognosis of patients with primary large B-cell lymphoma of the central nervous system（PCNSLBCL）.

**Methods:**

Clinical and pathologic data of 70 patients with PCNSLBCL admitted to Ruijin Hospital Affiliated to Shanghai Jiao Tong University School of Medicine from December 2010 to November 2022 were collected for retrospective analysis. Survival analysis was performed using the Kaplan-Meier method and log-rank test, and prognosis analysis was conducted using the Cox proportional hazards model.

**Results:**

Among 70 patients with PCNSLBCL, complete remission（CRs）were achieved in 49（70.0％）and partial remission in 4（5.7％）after the first-line induction therapy; the overall remission rate was 75.7％. The 2-year progression-free survival（PFS）rate was 55.8％ and the median progression-free survival（mPFS）time was 35.9 months, whereas the 2-year overall survival（OS）rate was 79.1％ with a median OS time not reached. After CR induced by first-line therapy, cumulative incidence of relapse（CIR）was lower in patients who had received auto-HSCT than in those who had not received consolidation therapy（*P*＝0.032）, whose 2-year PFS rate was 54.4％ and mPFS time was 35.9 months; comparatively, the 2-year PFS rate in patients having received oral maintenance of small molecule drugs reached 84.4％ with a mPFS time of 79.5 months（*P*＝0.038）. Multivariant analysis demonstrated that Class 3 in the Memorial Sloan-Kettering Cancer Center（MSKCC）prognostic model is an independent adverse prognostic factor of OS in patients with PCNSLBCL（*HR*＝3.127, 95％ *CI* 1.057–9.253, *P*＝0.039）.

**Conclusion:**

In patients with PCNSLBCL achieving CR after the first-line induction therapy, auto-HSCT as consolidation therapy would lead to a decreased CIR, and PFS time could be prolonged by oral maintenance of small molecule drugs. Class 3 MSKCC prognostic model is independently associated with poorer OS.

原发中枢神经系统淋巴瘤是一类以脑实质、脊髓、眼、颅神经和（或）脑膜等结外部位受累为首发表现的特殊类型淋巴瘤，占颅内肿瘤的3％～4％、结外淋巴瘤的4％～6％[1]–[2]，其中95％形态学为弥漫大B细胞淋巴瘤（DLBCL）[3]，即原发中枢神经系统大B细胞淋巴瘤（PCNSLBCL），其预后异质性强[4]。国内外指南、专家共识[3],[5]–[7]对于PCNSLBCL的诱导治疗均推荐以大剂量甲氨蝶呤（HD-MTX）为基础联合或不联合利妥昔单抗的方案；巩固治疗方面，auto-HSCT与全脑放疗均得到推荐，但后者因远期神经毒性（尤其在年龄>60岁的患者中）其临床应用受到限制。小分子药物在临床实践中已有探索性尝试[8]–[9]，安全性与有效性仍有待大规模、前瞻性临床研究验证。本研究对70例PCNSLBCL患者进行回顾性分析，旨在探究其临床病理特点及不同治疗方案的预后差异。

## 病例与方法

1. 病例：将2010年10月至2022年11月上海交通大学医学院附属瑞金医院收治的70例PCNSLBCL患者纳入研究。所有患者均经脑活组织病理检查和免疫组织化学（IHC）染色确诊为DLBCL，参照WHO淋巴组织肿瘤分类2016年修订版复核，并通过影像学检查确认受累范围局限于中枢神经系统和（或）眼。患者预后分层参照纪念斯隆-凯特琳肿瘤中心（MSKCC）预后模型[10]，50岁及以下者为1类，若Karnofsky体能评分≥70分（生活可完全自理）则为2类，否则为3类。双表达定义为c-Myc阳性率≥40％且Bcl-2阳性率≥50％[11]。

2. 治疗方案：70例患者中，68例接受了含HD-MTX方案的诱导治疗，5例患者因治疗期间出现急性肾损伤或者其他不能耐受的情况，后续疗程将HD-MTX更换为替莫唑胺。20例患者在HD-MTX基础上联合应用小分子药物，其中3例联用来那度胺，16例联用布鲁顿酪氨酸激酶（BTK）抑制剂（伊布替尼2例、泽布替尼7例和奥布替尼7例），1例同时联用来那度胺和泽布替尼。2例高龄（>80岁）患者接受了不含HD-MTX方案的诱导治疗，其中1例接受ZR^2^（泽布替尼+利妥昔单抗+来那度胺）无化疗方案，1例接受单纯放疗。

49例一线诱导治疗取得完全缓解（CR）的患者中，13例接受auto-HSCT作为巩固治疗，移植前2例采用BuCyE（白消安+环磷酰胺+依托泊苷）方案预处理，8例采用BEAM（卡莫司汀+依托泊苷+阿糖胞苷+美法仑）方案预处理，3例采用TT-BCNU（噻替派+卡莫司汀）方案预处理；18例口服小分子药物维持6个月及以上作为巩固治疗，其中15例服用BTK抑制剂（伊布替尼6例、泽布替尼9例），3例服用来那度胺；接受全脑放疗或立体定向放疗作为巩固治疗的有3例；未接受任何巩固治疗的有15例。

3. 疗效评估：一线诱导治疗结束后通过头颅增强磁共振成像（MRI）和（或）PET-CT按照国际原发中枢神经系统淋巴瘤合作组（IPCG）标准[12]评估疗效，分为CR、部分缓解（PR）、疾病稳定（SD）和疾病进展（PD），客观缓解率（ORR）以CR率与PR率之和计算。

4. 随访：采用电话、微信回访及病史记录查阅等形式随访，随访截止日期为2023年10月30日，中位随访时间为32.9个月。总生存（OS）期定义为自初次接受治疗当日至全因死亡或末次随访的时间间隔，无进展生存（PFS）期定义为自初次接受治疗当日至疾病进展、首次复发或末次随访的时间间隔。

5. 统计学处理：采用R 4.3.1软件分析数据。计数资料以频数（％）表示，组间比较采用Fisher确切概率法；生存分析采用Kaplan-Meier法及Log-rank检验，预后因素分析采用Cox比例风险模型，单因素分析结果*P*<0.10者纳入多因素分析。以*P*<0.05为差异具有统计学意义。

## 结果

1. 临床和病理特征：70例患者的男女比例为1.26:1，中位起病年龄59（23～83）岁，其中50岁及以下的患者占20.0％，65岁以上的患者占30.0％，Karnofsky体能评分<70分（生活不能完全自理）的患者占38.6％，有深部受累（包括基底节、胼胝体、脑干、小脑）的患者占45.7％，病灶数量≥2处的患者占64.3％，血清LDH升高的患者占48.6％，MSKCC预后评分1、2、3类分别占20.0％、51.4％、28.6％。患者初发时均出现神经精神症状，最常见的临床表现为运动障碍（41.4％），其次是头痛（31.4％）和意识障碍（24.3％）（表1）。1例患者同时主诉烦渴、多尿。所有患者均未诉B症状。

**表1:** 70例原发中枢神经系统大B细胞淋巴瘤患者初发时的神经精神症状

症状	例（%）
运动障碍（肢体、颜面、舌、咽肌力下降）	29（41.4）
头痛	22（31.4）
意识障碍	17（24.3）
意识内容改变（意识模糊、谵妄）	13（18.6）
觉醒度改变（嗜睡）	9（12.9）
头晕（排除眩晕）	16（22.9）
恶心和（或）呕吐	12（17.1）
颅内压升高	11（15.7）
前庭功能障碍	1（1.4）
认知障碍（记忆障碍、失语）	11（15.7）
视觉障碍（视力障碍、视野缺损、复视）	9（12.9）
感觉障碍	4（5.7）
癫痫发作	4（5.7）
听觉障碍（耳鸣）	3（4.3）
眩晕	2（2.9）
尿便障碍	2（2.9）
共济失调	1（1.4）
性格改变	1（1.4）

病理特征方面，66例患者的免疫组织化学染色结果可根据Hans分类确定细胞起源，其中41例为非生发中心B细胞（GCB）亚型；41例患者有c-Myc和Bcl-2的半定量结果，其中双表达16例（39.0％）。在双表达PCNSLBCL患者中，MSKCC预后评分1类、2类、3类分别占6.2％、50.0％、43.8％，而在非双表达患者（25例）中分别为32.0％、52.0％、16.0％。有FISH检测结果记录的患者中，1例检出MYC重排（1/48），均未检出BCL2重排（0/47），5例检出BCL6重排（5/47），均非双打击［定义为MYC重排伴BCL2和（或）BCL6重排］，1例检出IRF4重排（1/19），2例检出11q异常（均有11q23.3扩增，1例同时检出11q24.3缺失），5例检出TP53缺失（5/25）。

29例患者的肿瘤组织经二代测序检测，突变频率较高的基因有IGLL5（82.8％）、PIM1（79.3％）、MYD88（69.0％，均为p.L265P）、KMT2D（55.2％）、CD79B（51.7％），其中MYD88、CD79B共突变9例（31.0％）（图1）。

**图1:**
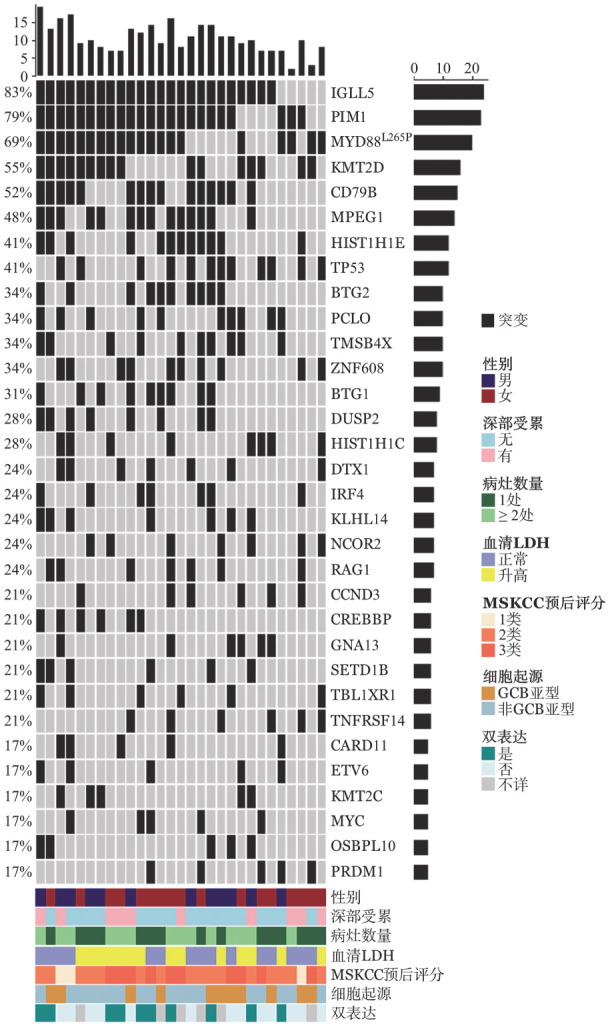
29例原发中枢神经系统大B细胞淋巴瘤患者的基因突变谱 **注** MSKCC：纪念斯隆-凯特琳肿瘤中心；GCB：生发中心B细胞

2. 疗效和生存分析：70例患者经一线诱导治疗后，49例（70.0％）评价为CR，4例（5.7％）评价为PR，1例（1.4％）评价为SD，16例（22.9％）评价为PD，ORR为75.7％。一线诱导治疗加入口服小分子靶向药物的21例患者中，15例（71.4％）评价为CR，2例（9.5％）评价为PR，ORR高于未口服小分子靶向药物的患者，但差异无统计学意义（81.0％对73.5％，*P*＝0.20）。

70例患者2年PFS率55.8％，中位PFS期为35.9个月；2年OS率为79.1％，中位OS期未达到。一线诱导治疗后CR患者OS优于未CR患者（*P*<0.001）：CR患者（49例）2年OS率为93.7％，中位OS期未达到；未达到CR患者（21例）2年OS率为40.4％，中位OS期为20.4个月（图2）。

**图2:**
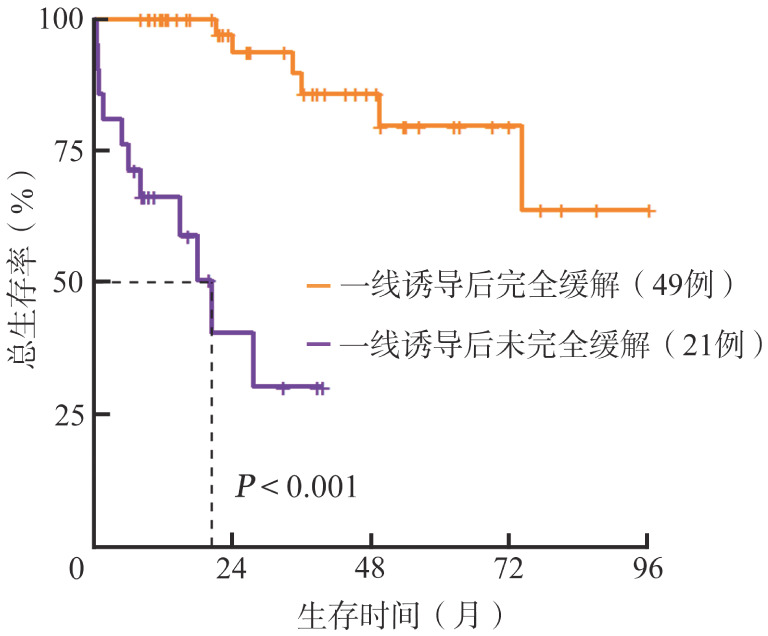
原发中枢神经系统大B细胞淋巴瘤患者一线诱导治疗后总生存曲线

在49例一线诱导治疗后CR的患者中，接受auto-HSCT作为巩固治疗的患者（13例）累积复发风险低于未接受巩固治疗的患者（15例），2年累积复发率分别为9.1％和45.6％（*P*＝0.032，图3）。另外，口服小分子药物维持巩固的患者（21例）PFS优于无巩固治疗的患者（15例），2年PFS率分别为84.4％和54.4％，中位PFS期分别为79.5个月和35.9个月（*P*＝0.038，图4）。

**图3:**
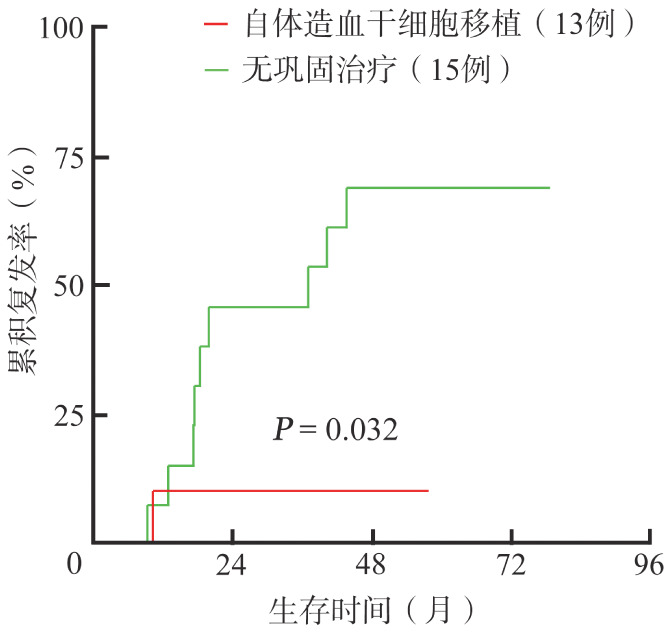
自体造血干细胞移植巩固治疗以及无巩固治疗原发中枢神经系统大B细胞淋巴瘤患者的复发曲线

**图4:**
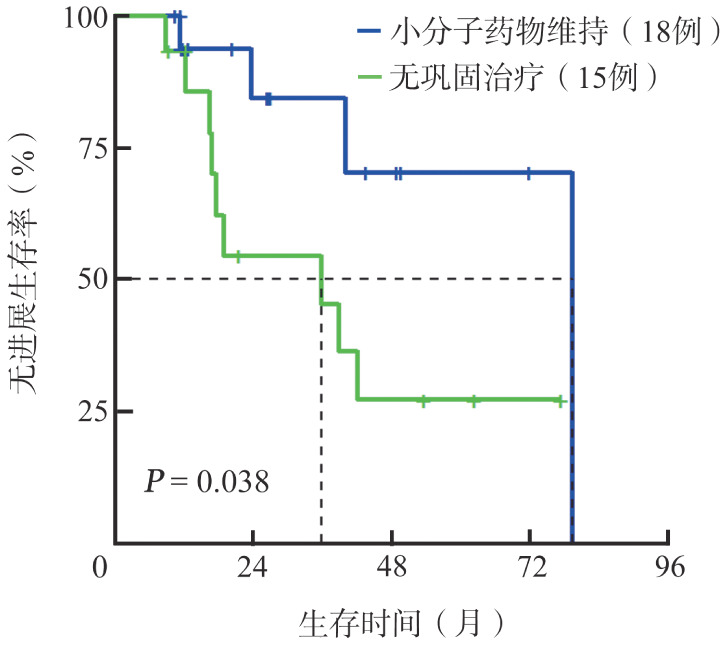
小分子药物巩固治疗以及无巩固治疗原发中枢神经系统大B细胞淋巴瘤患者的无进展生存曲线

在41例可评价肿瘤组织c-Myc、Bcl-2双表达情况的患者中，双表达者一线诱导治疗后CR率50.0％（8/16），非双表达者一线诱导治疗后CR率76.0％（19/25），差异无统计学意义（*P*＝0.065）。8例双表达且一线诱导治疗后CR的患者：1例采用HD-MTX联合奥布替尼的方案并序贯auto-HSCT，PFS期为22.3个月，目前未复发，1例采用HD-MTX联合泽布替尼、来那度胺的方案且未接受巩固治疗，PFS期为21.4个月，目前未复发，6例口服小分子药物维持患者的中位PFS期短于5例口服小分子药物维持巩固的非双表达患者（23.6个月对未达到，*P*＝0.034，图5）。

**图5:**
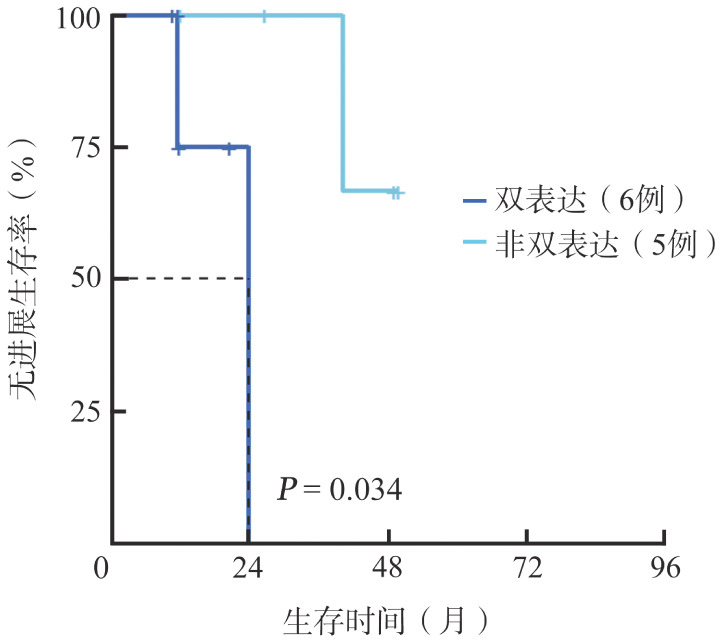
双表达及非双表达原发中枢神经系统大B细胞淋巴瘤患者的无进展生存曲线 **注** 双表达：c-Myc阳性率≥40％且Bcl-2阳性率≥50％

3. 预后因素分析：如表2所示，将性别、年龄、Karnofsky体能评分、深部受累、病灶数量、血清LDH、MSKCC预后评分、细胞起源情况等临床和病理特点纳入单因素分析，结果显示非GCB亚型细胞起源与较差的OS相关（*HR*＝4.931，95％*CI* 1.097～22.170，*P*＝0.038）。将血清LDH、MSKCC预后评分、细胞起源纳入多因素分析，结果显示MSKCC预后评分3类是影响OS的独立不良预后因素（*HR*＝3.127，95％*CI* 1.057～9.253，*P*＝0.039）。

**表2:** 影响原发中枢神经系统大B细胞淋巴瘤患者预后的单因素和多因素分析

变量	无进展生存期单因素分析		总生存期			
			单因素分析		多因素分析	
	*HR*（95% *CI*）	*P*值	*HR*（95% *CI*）	*P*值	*HR*（95% *CI*）	*P*值
女性	1.468(0.763～2.825)	0.250	1.476(0.567～3.838)	0.425		
深部受累	0.744(0.383～1.447)	0.384	1.660(0.631～4.368)	0.304		
病灶数量≥2处	0.940(0.480～1.843)	0.858	1.499(0.528～4.257)	0.448		
LDH升高	1.594(0.825～3.079)	0.166	2.433(0.893～6.628)	0.082	1.842(0.596～5.694)	0.289
MSKCC预后评分3类	1.159(0.540～2.489)	0.704	2.564(0.957～6.870)	0.061	3.127(1.057～9.253)	0.039
细胞起源非GCB亚型	1.870(0.884～3.955)	0.102	4.931(1.097～22.170)	0.038	3.848(0.837～17.690)	0.083

**注** MSKCC：纪念斯隆-凯特琳肿瘤中心；GCB：生发中心B细胞

## 讨论

PCNSLBCL是一种少见的结外侵袭性淋巴瘤类型，临床表现与具体发病部位（脑实质、软脑膜、眼、脊髓等）相关，而B症状少见（2％）[13]。本研究纳入的PCNSLBCL患者首发表现以运动障碍、头痛、意识内容和（或）觉醒度改变最为常见，无B症状记录。

免疫表型方面，国内外PCNSLBCL报道中非GCB亚型比例差异较大。国内一项研究报道的比例为64.6％（73/113）[14]，与本文所报道的60.6％相近，而其他报道中非GCB亚型的比例可达77.1％～96.3％[15]–[18]；本文报道c-Myc、Bcl-2双表达占39.0％，与既往文献报道的30.6％～35.4％相近[19]–[20]。

治疗方面，以HD-MTX为基础的一线诱导治疗给PCNSL患者带来明确获益，但对于联合药物尚无共识[21]。相较于HD-MTX单药诱导治疗初治PCNSL患者，联合更多化疗药物可加深缓解，ORR由既往35％提升到70％以上[22]。IELSG32研究显示MATRix（甲氨蝶呤+阿糖胞苷+噻替哌+利妥昔单抗）方案具有优于甲氨蝶呤、阿糖胞苷联合或不联合利妥昔单抗方案的疗效，ORR为79％[23]，但不良反应尤其是血液学毒性较大，4级中性粒细胞缺乏发生率为56％，4级发热性中性粒细胞缺乏发生率为1％，4级血小板减少发生率为73％，4级贫血发生率为5％，均高于R-MA（利妥昔单抗+甲氨蝶呤+阿糖胞苷）方案[24]，提示联合化疗诱导模式下疗效和安全性的平衡仍值得考量。本文报道的70例PCSNLBCL患者接受的一线诱导治疗为HD-MTX或者替莫唑胺（MTX不耐受患者），ORR为75.7％，与之相近，其中联合应用小分子药物的患者ORR可达81.0％（17/21），提示小分子药物在一线诱导方案中也可供选择，其安全性有待探索。IELSG研究中MATRix组（无论是auto-HSCT还是全脑放疗巩固）2年PFS率和OS率分别为61.0％、69.0％，而在本研究中接受小分子药物维持巩固治疗的患者2年PFS率和OS率分别为84.4％、100％，提示口服小分子药物维持同样可以作为巩固治疗的选择，但随访时间较短，尚不清楚远期生存获益是否更优，仍有待进一步验证。

auto-HSCT是淋巴瘤治疗的重要方法[25]，在PCNSLBCL的巩固治疗中也得到一线推荐[5]。本研究中13例患者在一线诱导治疗CR后行auto-HSCT，移植后复发1例，无复发死亡1例，累积复发风险低于未接受巩固治疗的患者，提示auto-HSCT仍是可耐受移植患者降低复发风险的重要选择。同时口服小分子药物维持治疗相较于无巩固治疗，患者的PFS期延长，因此可作为老年或不耐受移植的患者巩固治疗的选择。

PCNSLBCL的预后异质性强，总体较差。在本研究中MSKCC预后评分3类仍是影响OS的独立不良预后因素，而对PFS影响无统计学意义，说明患者年龄和体能状态评分对患者的生存有重要的影响，小分子药物维持等巩固治疗有效减少复发，延长了患者的PFS期。

在DLBCL中，MYD88 p.L265P、CD79B突变及其他相关基因突变导致B细胞受体（BCR）通路及下游的NF-κB通路的持续激活，而BTK是BCR/NF-κB通路交流的关键蛋白，其抑制剂已取得肯定的疗效[26]，且能有效通过血脑屏障[27]，为PCNSLBCL的治疗提供了新的选择。已有小规模临床研究探索并肯定了伊布替尼在PCNSLBCL中的疗效[28]–[31]，泽布替尼、奥布替尼的应用多为病例报道[32]–[35]，有待积累更多循证医学证据。双表达PCNSLBCL患者CR率较低，预后更差，与既往报道一致[36]–[37]。Kim等[38]报道双表达PCNSLBCL（采用的双表达标准更宽松，c-Myc阳性率≥40％且Bcl-2阳性率≥30％）与更高的MSKCC预后评分有关。在本研究中，双表达PCNSLBCL患者与非双表达者MSKCC 2类占比相近，分别为50.0％（8/16）和52.0％（13/25），但双表达患者MSKCC 3类占43.8％（7/16），而在非双表达患者中为16.0％（4/25）。更高的MSKCC 3类比例可以部分解释双表达患者较短的OS期，但是在一线诱导CR后小分子药物维持巩固的患者中，双表达仍造成更短的PFS期，耐药或其他潜在造成复发的机制有待探索。

综上所述，PCNSLBCL是一种罕见的侵袭性结外非霍奇金淋巴瘤，MSKCC 3类患者OS期更短，auto-HSCT巩固治疗显著降低复发风险，以BTK抑制剂为代表的小分子药物维持治疗可改善PCNSLBCL患者的预后，但在双表达患者中疗效仍欠佳，未来需要开展多中心、大样本、前瞻性的临床研究进一步探索。
